# Prognostic Value of Cancer Stem Cell Markers in Head and Neck Squamous Cell Carcinoma: a Meta-analysis

**DOI:** 10.1038/srep43008

**Published:** 2017-02-21

**Authors:** Zhaona Fan, Mianxiang Li, Xiaobing Chen, Juan Wang, Xueyi Liang, Hongfei Wang, Zhi Wang, Bin Cheng, Juan Xia

**Affiliations:** 1 Guangdong Provincial Key Laboratory of Stomatology, Guanghua School of Stomatology, Sun Yat-sen University, Guangzhou, Guangdong 510055, China

## Abstract

Bmi-1, CD133, Nanog and Oct-4 have been reported as cancer stem cell (CSC) markers in head and neck squamous cell carcinoma (HNSCC). However, the prognostic value of them in HNSCC remains controversial. Hence, this meta-analysis was conducted to access the association between the four CSC markers and survival outcome of HNSCC patients. A total of 22 articles with 27 studies met the inclusion criteria and the combined hazard ratio (HR) and 95% confidence intervals (95% CI) were calculated. Data analysis showed that high expression of CSC markers was associated with poor overall survival (OS) (HR = 1.93; 95% CI: 1.46–2.55, *P* < 0.001) and disease free survival (DFS) (HR = 4.78; 95% CI: 2.95–7.75, *P* < 0.001) but not disease specific survival (DSS) (HR = 1.17; 95% CI: 0.74–1.84, *P* = 0.50) of HNSCC patients. Subgroup analysis indicted that high expression of CD133 (HR = 2.33, 95%CI: 1.42–3.83, *P* < 0.001), Oct-4(HR = 2.10, 95%CI: 1.36–3.22, *P* = 0.007) and Nanog (HR = 2.49, 95%CI: 1.66–3.72, *P* < 0.001) could predict poor OS in HNSCC patients respectively whereas overexpression of Bmi-1 was not related to the reduced OS in HNSCC patients (HR = 1.32, 95%CI: 0.66–2.65, *P* = 0.43). Therefore, we concluded that CSC markers, especially CD133, Nanog and Oct-4, might be predictive factors in HNSCC patients.

Head and neck squamous cell carcinoma (HNSCC) is the most common histological type of head and neck cancer which is the sixth leading cancer by incidence worldwide, leading to more than 200,000 deaths annually^1^,^2^. Although treatments for HNSCC have been progressing rapidly recently, the overall survival of patients with HNSCC is relatively low because the regional and distant metastases are already existed at diagnosis. More seriously, the five-year survival rate of HNSCC on the whole is lower than 50%^3^,^4^.

Accumulated evidence suggests that cancer stem cells (CSCs) may played an important role in the progression and prognosis of cancers, including HNSCC^5^. CSCs, a small subpopulation of cancer cells, possess the ability to initiate neoplasm and sustain tumor self-renewal^6^. Several stem cell markers have been described for HNSCC, such as CD44, Bmi-1, CD133, ALDH1, Nanog, Oct-4 and SOX2^7^,^8^,^9^. Furthermore, it has been reported that patients with high expression of ALDH1, CD44 and SOX2 had worse prognosis^10^,^11^,^12^. However, due to differences in research method, study population and sample size, the role of Bmi-1, CD133, Nanog and Oct-4 in HNSCC is still not clear to date. There are conflicting opinions about their prognostic value.

In the present study, we collected the available literatures and conducted this meta-analysis to combine the evidence of CSC markers (CD133, Nanog, Bmi-1, Oct-4) in patients with HNSCC, in order to address controversial issues.

## Results

### Study characteristics

**Figure 1:**
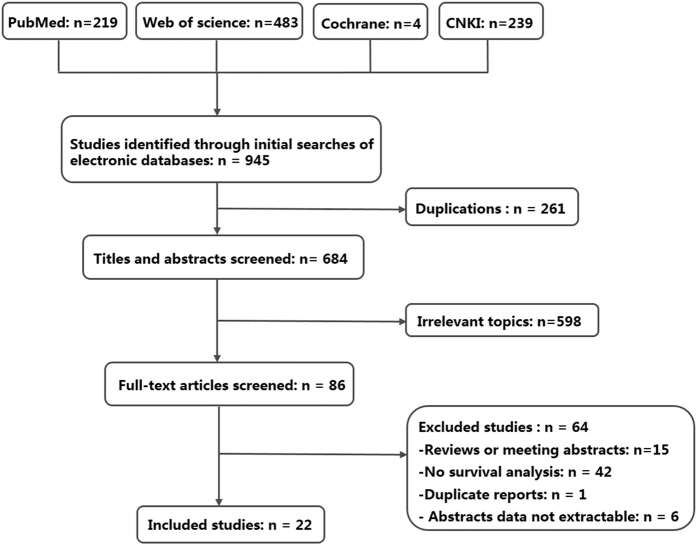
Flow diagram of studies identified, included, and excluded.

The literature selection process of the eligible studies was presented in Fig. 1. A total of 22 articles^13^,^14^,^15^,^16^,^17^,^18^,^19^,^20^,^21^,^22^,^23^,^24^,^25^,^26^,^27^,^28^,^29^,^30^,^31^,^32^,^33^,^34^ including 27 studies and 2143 patients met the inclusion criteria for the meta-analysis. The basic characteristics of each eligible studies were summarized in Table 1. All articles were published between 2006 and 2016, and the Newcastle–Ottawa Scale (NOS) scores of them were listed in Table 1 35. The majority of these studies were proceeded in Asia (n = 22), and others were conducted in Europe (n = 5). Among the 27 included studies, 9 studies involved patients with Bmi-1, 3 studies involved patients with CD133, 9 studies involved patients with Oct-4 and 6 studies involved patients with Nanog. The sample sizes of the included studies ranged from 50 to 436. According to the median of all samples, 14 studies were classified as large sample size (n > 72) studies and 13 studies were small sample size (n < 72) studies. Twenty-two studies explored the prognostic value of the four markers in overall survival (OS), whereas 9 studies investigated the prognostic significance of the four markers in disease free survival (DFS) or disease specific survival (DSS).

### CSC markers and OS in HNSCC

**Figure 2:**
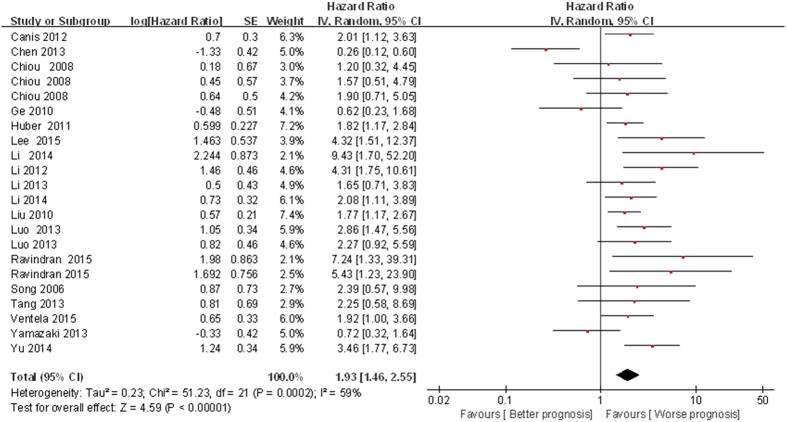
Forest plot of the association between CSC markers and OS in patients with HNSCC.

Twenty-two studies^14^,^15^,^16^,^18^,^20^,^21^,^24^,^26^,^27^,^28^,^29^,^30^,^31^,^32^,^34^ with 1759 patients reported the data of 4 CSC markers and OS in HNSCC. High expression of CSC markers was associated with poor OS (HR = 1.93; 95% CI: 1.46–2.55, *P* < 0.001) although with heterogeneity (I^2^ = 59%, *P*_*h*_ < 0.001; Fig. 2).

### CSC markers and DSS in HNSCC

**Figure 3:**
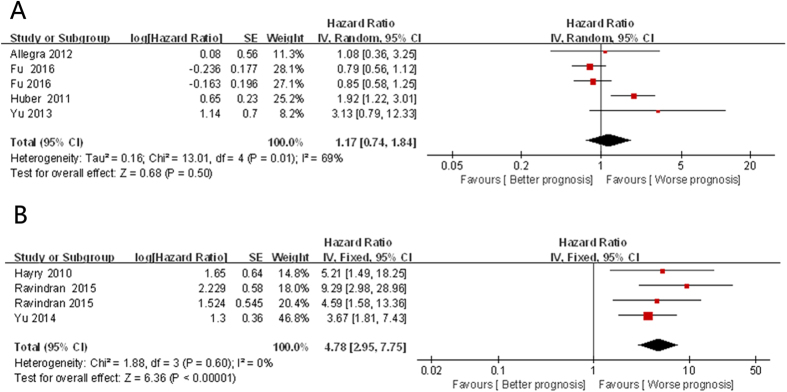
Forest plot of the association between CSC markers and DSS (**A**)/DFS (**B**) in patients with HNSCC.

Five studies^13^,^17^,^20^,^34^ with 1182 patients showed the information of the 4 CSC markers and DSS in HNSCC. Data analysis showed that there was no significant relation between the overexpression of CSC markers and DSS (HR = 1.17; 95% CI: 0.74–1.84, *P* = 0.50). However, there was obvious heterogeneity (I^2^ = 69%, *P*_*h*_ = 0.01; Fig. 3A).

### CSC markers and DFS in HNSCC

The association of the 4 CSC markers and DFS in HNSCC was supplied by 4^19^,^28^,^34^ studies with 276 patients. Data analysis showed that the overexpression of CSC markers was related to poor DFS (HR = 4.78; 95% CI: 2.95–7.75, *P* < 0.001) without obvious heterogeneity (I^2^ = 0%, *P*_*h*_ = 0.60; Fig. 3B).

### Subgroup Analysis

Subgroup analysis stratified by different CSC markers, ethnicity, sample size and tumor location were performed to detect the potential source of heterogeneity (Table 2). According to the stratification by different CSC markers, the finding of poor OS in patients with high expression of CSC markers was consistently found in CD133 (HR = 2.33, 95%CI: 1.42–3.83, *P* < 0.001; I^2^ = 22%, *P*_*h*_ = 0.28), Oct-4 (HR = 2.10, 95%CI: 1.36–3.22, *P* < 0.001; I^2^ = 40%, *P*_*h*_ = 0.11) and Nanog (HR = 2.49, 95%CI: 1.66–3.72, *P* < 0.001; I^2^ = 0%, *P*_*h*_ = 0.60) except Bmi-1(HR = 1.32, 95%CI: 0.66–2.65, *P* = 0.43; I^2^ = 81%, *P*_*h*_ < 0.001). Interestingly, poor DSS in patients was significantly associated with high expression of Bmi-1 (HR = 1.85, 95%CI: 1.24–2.76, *P* = 0.002; I^2^ = 0%, *P*_*h*_ = 0.47). There were consistent findings in Asian (HR = 1.96, 95%CI: 1.37–2.80, *P* < 0.001; I^2^ = 65%, *P*_*h*_ < 0.001) and Caucasian (HR = 1.89, 95%CI: 1.39–2.59, *P* < 0.001; I^2^ = 0%, *P*_*h*_ = 0.96) between CSC markers and OS. However, overexpression of CSC markers did not predict poor DSS in Asians (HR = 0.90, 95%CI: 0.61–1.34, *P* = 0.61, I^2^ = 45, *P*_*h*_ = 0.16). According to the subgroup analysis of tumor location, poor OS was related to CSC markers in oral/oropharyngeal squamous cell carcinoma (OSCC) (HR = 2.14, 95%CI: 1.40–3.26, *P* = 0.004; I^2^ = 19%, *P*_*h*_ = 0.29), laryngeal squamous cell carcinoma (LSCC) (HR = 3.18, 95%CI: 1.75–5.78, *P* < 0.001; I^2^ = 0%, *P*_*h*_ = 0.58) and nasopharyngeal carcinoma (NEPC) (HR = 2.60, 95%CI: 1.58–4.30, *P* < 0.001; I^2^ = 0%, *P*_*h*_ = 0.91) without esophageal squamous cell carcinoma (ESCC) (HR = 1.48, 95%CI: 0.70–3.12, *P* = 0.30; I^2^ = 83%, *P*_*h*_ < 0.001). When it comes to the sample size, there was no significance between CSC markers and poor OS in large sample size (HR = 1.49, 95%CI: 0.97–2.30, *P* = 0.07, I^2^ = 75, *P*_*h*_ < 0.001) but in small sample size (HR = 2.41, 95%CI: 1.81–3.22, *P* < 0.001, I^2^ = 3, *P*_*h*_ = 0.41).

### Sensitivity analysis and Publication Bias

Seventeen studies^13^,^14^,^15^,^17^,^18^,^19^,^20^,^21^,^22^,^23^,^24^,^25^,^26^,^28^,^29^,^30^,^34^ that scored seven or more on the NOS were included in sensitivity analysis (Table 3).There was no change in the significance of most of the results except the relation between Oct-4 and OS (HR = 2.14, 95%CI: 0.97–4.74, *P* = 0.06, I^2^ = 62, *P*_*h*_ = 0.03), LSCC and OS (HR = 2.25, 95%CI: 0.58–8.69, *P* = 0.24), and NPEC and OS (HR = 2.39, 95%CI: 0.57–9.98, *P* = 0.23), which was shown to be no significance.

**Figure 4:**
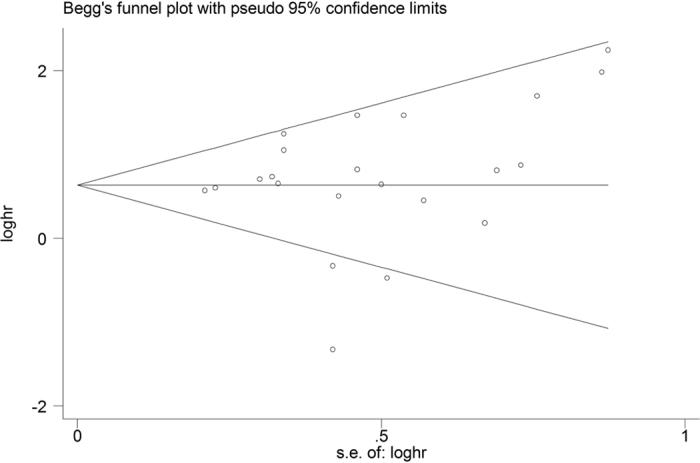
Begg’s funnel plot of publication bias test for OS in HNSCC.

We performed a Begg’s funnel plot with using Begg’s test and Egger’s test to assess the publication bias of the included studies of OS. As showed in Fig. 4, there was no obvious publication bias in these studies (Begg’s test: *P* = 0.120; Egger’s test: *P* = 0.500).

## Discussion

This meta-analysis of 27 studies including 2143 patients assessing the prognostic value of 4 CSC markers in HNSCC showed that a high expression level of CSC markers was a promising prognostic factor for lower DFS and OS in HNSCC patients. However, the expression of CSC markers had no obvious influence on DSS of HNSCC patients. According to the current results, CSC markers may play an important role in the relapse of HNSCC rather than death from HNSCC. However, considering that the sample sizes about DSS and DFS are relatively limited, our results need to be cautiously interpreted.

Bmi-1 was related to multiple human cancers which played an indispensable role in maintaining the self-renewal ability of both normal and malignant cancer stem cells. It has been reported that high expression of Bmi-1 could significantly lead to a poor OS in gastric cancer patients^36^. But in our subgroup analyses, Bmi-1 could not influence the OS of HNSCC patients. However, poor DSS in HNSCC patients was significantly associated with high expression of Bmi-1 with no heterogeneity (I^2^ = 0%, *P*_*h*_ = 0.47). It is worth noting that there was high heterogeneity between OS and HNSCC (I^2^ = 81%, *P*_*h*_ < 0.001). We tried to find what caused this. When we ruled out the study of Chen 2013^15^, the association of poor OS and high expression of Bmi-1 was changed to be significant, meanwhile the heterogeneity was lower than before. This results suggested that we need more studies to get a conclusion whether the expression of Bmi-1 can influence the OS of HNSCC patients.

CD133, also named prominin-1, has been verified to be a CSC marker in many cancers. Previous meta-analyses showed that high expression of CD133 was responsible for the reduced OS of ovarian cancer, gastric cancer, non-small cell lung cancer and hepatocellular carcinoma patients^37^,^38^,^39^,^40^. Our subgroup analysis revealed that a high expression of CD133 was related to a lower OS in HNSCC patients which was in keeping with those previous analyses.

Nanog and Oct-4, also known as embryonic stem cells markers, both have the ability in maintaining the self-renewal capacity in embryonic stem cells^28^. Moreover, studies have showed that the co-expression of Oct-4 and Nanog could be found in hepatocellular carcinoma and lung adenocarcinoma^41^,^42^. Our results suggested that the high expression of Nanog and Oct-4 could reduce the OS of HNSCC patients, respectively. In the research of Chiou *et al*.^16^, patients with high expression of both Nanog and Oct-4 were associated with lower OS than those with high expression of Nanog or Oct-4 alone^16^. This reminded us that more studies about the relationship of co-expression of Nanog and Oct-4 and the survival outcome of HNSCC should be conducted.

In the subgroup analysis of tumor location, poor OS was related to CSC markers in OSCC, LSCC and NEPC except ESCC. Interestingly, when we tried to find the origin of the heterogeneity in CSC markers and ESCC, we found that, as same as the result of the association of Bmi-1 and OS, the heterogeneity was lower and the result was significantly changed (HR = 2.03, 95%CI: 1.47–2.82, *P* < 0.001, I^2^ = 10, *P*_*h*_ = 0.34) if we discarded the study of Chen 2013^15^. Taking this into consideration, we could not simply conclude that poor OS was not related to high expression of CSC markers in ESCC because the number of included studies was a little small and we needed more powerful evidences.

Considering the subgroup of ethnicity, the OS of the Asian and Caucasian was the same to the overall OS. The DSS of the Asian was consistent with the overall DSS while the DSS of the Caucasian was changed to be significant without heterogeneity. In the subgroup analysis of sample size, we used the median as the boundary because most of the sample size of our included studies were relatively small. As showed in Table 2, poor OS of large sample size was not significantly associated with high expression of CSC markers with high heterogeneity because Chen 2013^15^ was in this group. Without study of Chen 2013^15^, the results would be changed (HR = 2.09, 95%CI: 1.68–2.60, *P* < 0.001, I^2^ = 30, *P*_*h*_ = 0.10).

This meta-analysis has the following limitations that must be taken into consideration. First, both the number of included studies about each CSC markers and the number of included HNSCC patients in each study are relatively small, which, to some extent, may reduce the power and precision of our subcategory analyses. Second, most of the included studies were conducted in Asian and a few studies were about the Caucasians but no studies were about the black populations, which may produce potential population selection bias. Third, nonuniform cut-off value defining high and low expression of CSC markers may impact the results of this meta-analysis. Despite these limitations, we provided a comprehensive analysis of the association between CSC markers and OS/DFS/DSS of HNSCC patients. To the best of our knowledge, this meta-analysis is the first to systematically assess the association of Bmi-1, Oct-4, Nanog and CD133 expression with survival outcome of HNSCC. So far, there are some meta-analyses showed that high expression of CSC markers, including ALDH1, CD44 and SOX2, could predict poor OS/DFS in head and neck cancer patients^10^,^11^,^12^. Our results were in line with these previous analyses.

In summary, our meta-analysis revealed that high expression of CSC markers was significantly associated with poor OS and DFS but not DSS of HNSCC patients. However, because of certain limitations, different subgroup showed to some extent inconsistent results, which prompted future large-sample, well-designed with long-term follow-up to confirm and update the findings of this. Nevertheless, our study still gave some hints that CSC markers have prognostic value in HNSCC patients.

## Methods

### Literature search and eligibility Criteria

The databases of PubMed, Web of Science, the Cochrane library and China National Knowledge Infrastructure (CNKI) were thoroughly searched until July, 2016 without language restriction. The search strategy was listed as follows: (CD133 OR Bmi-1 OR Oct-4 OR Nanog) and (head and neck squamous cell carcinoma OR HNSCC OR ((oral OR laryn* OR pharyn* OR tongue OR oropharyn* OR nasopharyn* OR hypopharyn* OR trachea OR laryngopharyn* OR cervical tracheal OR cervical esophagus) AND (cancer* OR tumor* OR carcinoma* OR neoplasm*))). Two reviewers (F. Z. N and L. M. X) inspected all candidate articles independently. Discrepancies were resolved in consensus.

The inclusion criteria were: 1) the diagnosis of HNSCC was made based on pathological examination; 2) the expression of CD133 or Bmi-1 or Oct-4 or Nanog with OS/DSS/DFS about HNSCC was reported; 3) HRs and 95% CIs were provided in text or sufficient data was provided for the calculation of HRs and 95% CIs; 4) articles published as original research. In order to avoid duplicate inclusion of data, we selected only the more recent or complete article when multiple reports described the same population.

The exclusion criteria were:1) reviews, meeting abstracts, letters; 2) nonhuman studies; 3) sample size <50 patients.

### Data extraction

Two reviewers (C. X. B and W. J) independently extracted the following information from included studies: author, year of publication, study country, sample size, clinicopathological parameters, cut-off value of CD133 or Bmi-1 or Oct-4 or Nanog, survival data and the tumor location. Disagreements were resolved by discussion.

### Quality assessment

Two reviewers (L. X. Y and W. H. F) independently assessed the quality of included studies by the NOS^35^. Studies with NOS scores of more than 7 were defined as high quality. Consensus was reached by discussion when there were inconsistent results.

### Statistical Analysis

Hazard ratio (HR) was used as a summary statistic for survival outcomes as described by Parmar *et al*.^43^. An HR greater than 1 represented poor prognosis in HNSCC. Heterogeneity among primary studies was assessed by the Cochrane’s Q statistic and I^2^ statistic. Cochran Q test’ *P* value < 0.10 or I^2^ > 50% indicated large heterogeneity between studies and we used random effects models to calculate the pooled HR and 95% CI. Otherwise, the fixed effects model was used. We used the median as the boundary between the large and small sample size. Studies with sample size >72 were regarded as large sample size, otherwise was considered as small sample studies. Subgroup analyses stratified by different stem cell marker, ethnicity, sample size and tumor location were carried out. Sensitivity analysis were applied to high-quality studies (NOS > 6). The Begg’s funnel plots were used to evaluate publication bias. All statistical analysis were performed by using Review manager 5.3 software (Cochrane Collaboration, Oxford, UK) and Stata 12.0 statistical software (Stata Corporation, College Station, TX, USA). A two-tailed *P* < 0.05 was considered statistically significant.

## Additional Information

**How to cite this article**: Fan, Z. *et al*. Prognostic Value of Cancer Stem Cell Markers in Head and Neck Squamous Cell Carcinoma: a Meta-analysis. *Sci. Rep.* **7**, 43008; doi: 10.1038/srep43008 (2017).

**Publisher's note:** Springer Nature remains neutral with regard to jurisdictional claims in published maps and institutional affiliations.

## Figures and Tables

**Table 1:** Characteristics of included studies.

Author year	CSC marker	Country	Ethnicity	Tumor location	Follow-up (months)	Sample size	Gender (M/F)	Detection method	TMN stage	Cut-off value	Outcome	Hazard ratio	Study design	NOS score
Allegra 2012^13^	Bmi-1	Italy	Caucasian	LSCC	36 ± 21.5	64	58/6	IHC	II–IV	Score ≥ 10(0–15)	DSS	E	P	7
Canis 2012^14^	CD133	UK	Caucasian	HNSCC	NA	98	85/13	IHC	I–IV	>0	OS	E	P	7
Chen 2013^15^	Bmi-1	China	Asian	ESCC	60	80	57/23	IHC	I–IV	>5%	OS	E	P	8
Chiou 2008^16^	Oct-4 Nanog CD133	China	Asian	OSCC	2–65	52	NA	IHC	I–IV	>0	OS	E	P	6
Fu 2016^17^	Oct-4 Nanog	China	Asian	HNSCC	48.5	436	402/34	IHC	I–IV	Score ≥ 2 (0–7)	DSS	R	P	9
Ge 2010^18^	Oct-4	China	Asian	HNSCC	52 (7–69.5)	85	84/1	IHC	I–IV	Score ≥ 4 (0–7)	OS	E	P	9
Hayry 2010^19^	Bmi-1	Finland	Caucasian	HNSCC	>24	73	36/37	IHC	I–II	>50%	DFS	R	P	8
Huber 2011^20^	Bmi-1	Switzerland	Caucasian	OSCC	81	149	98/51	IHC	I–IV	Score ≥ 10(0–15)	OS DSS	R	P	7
Lee 2015^21^	Nanog	Korea	Asian	OSCC	3–125	57	36/21	IHC	I–IV	Score ≥ 6(0–7)	OS	R	P	8
Li 2012^22^	Oct-4	China	Asian	ESCC	38.02	50	37/13	IHC	I–III	Score > 3(0–7)	OS	E	P	9
Li 2013^23^	Oct-4	China	Asian	ESCC	99	58	42/16	IHC	I–IV	≥ 5%	OS	E	P	7
Li 2014^24^	Nanog	China	Asian	ESCC	65	69	40/29	IHC	I–IV	>10%	OS	E	P	7
Li 2014^25^	Bmi-1	China	Asian	OSCC	42.3	52	28/24	IHC	I–IV	Score > 4(0–12)	OS	R	P	8
Liu 2010^26^	Bmi-1	China	Asian	ESCC	25	171	129/42	IHC	I–IV	Score > 4(0–16)	OS	E	P	8
Luo 2013^27^	Oct-4 Nanog	China	Asian	NPEC	60.1 (8–92)	122	92/30	IHC	I–IV	Score ≥ 6(0–9)	OS	E	P	6
Ravindran 2015^28^	Oct-4 Nanog	India	Asian	HNSCC	31.9 (14–48)	60	34/26	IHC	I–IV	>16%	OS DFS	R	P	8
Song 2006^29^	Bmi-1	China	Asian	NPEC	15–60	75	41/24	IHC	I–IV	≥ 10%	OS	E	P	7
Tang 2013^30^	Oct-4	China	Asian	LSCC	56 (12–84)	69	64/5	TMA IHC	I-IV	Score ≥ 2 (0–3)	OS	E	P	7
Ventela 2015^32^	Oct-4	Finland	Caucasian	HNSCC	NA	52	NA	IHC	NA	NA	OS	E	P	4
Yamazaki 2013^31^	Bmi-1	Japan	Asian	HNSCC	92.57 (7.30–131.87)	91	57/34	IHC	I-IV	>0	OS	E	P	6
Yu 2013^33^	Bmi-1	China	Asian	NPEC	3–60	97	65/32	IHC	I-IV	Score ≥ 8 ≥0–15)	DSS	E	P	8
Yu 2014^34^	CD133	China	Asian	LSCC	15–100	83	75/8	IHC	I-IV	Score ≥ 3 (0–12)	OS DFS	E	P	6

NA: not available; R: reported in text; E: estimated; M/F: male/female; P: prospective; CSC: cancer stem cell; OS: overall survival; DFS: disease free survival; DSS: disease specific survival; NOS: Newcastle–Ottawa Quality Assessment Scale; HNSCC: head and neck squamous cell carcinoma; OSCC: oral/oropharyngeal squamous cell carcinoma; ESCC: esophageal squamous cell carcinoma; LSCC: laryngeal squamous cell carcinoma; NPEC: nasopharyngeal carcinoma; cut-off value: the value that can be diagnosed as a positive/high expression of CSC markers; IHC: Immunohistochemistry; TMA: tissue microarrays.

**Table 2:** Results of meta-analysis for CSC markers on prognostic effect in HNSCC patients.

	Variable	Study NO.	Sample size	HR (95%CI)	*P* value	Heterogeneity	
						I^2^ (%)	*P* value
OS	Overall	22	1759	1.93 (1.46–2.55)	<0.001	59	<0.001
	Ethnicity						
	Asian	19	1460	1.96 (1.37–2.80)	<0.001	65	<0.001
	Caucasian	3	299	1.89 (1.39–2.59)	<0.001	0	0.96
	CSC markers						
	Bmi-1	6	618	1.32 (0.66–2.65)	0.43	81	<0.001
	CD133	3	233	2.33 (1.42–3.83)	<0.001	22	0.28
	Oct-4	8	548	2.10 (1.36–3.22)	<0.001	40	0.11
	Nanog	5	360	2.49 (1.66–3.72)	<0.001	0	0.60
	Tumor location						
	OSCC	6	414	2.14 (1.40–3.26)	0.004	19	0.29
	ESCC	5	428	1.48 (0.70–3.12)	0.30	83	<0.001
	LSCC	2	152	3.18 (1.75–5.78)	<0.001	0	0.58
	NPEC	3	319	2.60 (1.58–4.30)	<0.001	0	0.91
	Sample size						
	Large	10	1076	1.49 (0.97–2.30)	0.07	75	<0.001
	Small	12	683	2.41 (1.81–3.22)	<0.001	3	0.41
DSS	Overall	5	1182	1.17 (0.74–1.84)	0.50	69	0.01
	Ethnicity						
	Asian	3	969	0.90 (0.61–1.34)	0.61	45	0.16
	Caucasian	2	213	1.76 (1.16–2.68)	0.008	0	0.35
	Sample size						
	Large	2	872	0.82 (0.63–1.06)	0.12	0	0.78
	Small	3	310	1.85 (1.24–2.76)	0.002	0	0.47
	CSC markers						
	Bmi-1	3	310	1.85 (1.24–2.76)	0.002	0	0.47
	Oct-4	1	436	0.79 (0.56–1.12)	0.18	—	—
	Nanog	1	436	0.85 (0.58–1.25)	0.41	—	—
DFS	Overall	4	276	4.78 (2.95–7.75)	<0.001	0	0.60

CSC: cancer stem cell; OS: overall survival; DFS: disease free survival; DSS: disease specific survival; OSCC: oral/oropharyngeal squamous cell carcinoma; ESCC: esophageal squamous cell carcinoma; LSCC: laryngeal squamous cell carcinoma; NPEC: nasopharyngeal carcinoma.

**Table 3:** Sensitivity analysis of the meta-analysis results.

	Variable	Study NO.	Sample size	HR (95%CI)	P value	Heterogeneity	
						I^2^ (%)	P value
OS	Overall	14	1133	1.99 (1.34–2.97)	<0.001	68	<0.001
	Ethnicity						
	Asian	12	886	2.09 (1.24–3.54)	0.006	73	<0.001
	Caucasian	2	247	1.89 (1.32–2.69)	<0.001	0	0.79
	CSC markers						
	Bmi-1	5	527	1.52 (0.69–3.36)	0.30	83	<0.001
	CD133	1	98	2.01 (1.12–3.63)	0.02	—	—
	Oct-4	5	322	2.14 (0.97–4.74)	0.06	62	0.03
	Nanog	3	186	2.87 (1.62–5.07)	<0.001	13	0.32
	Tumor location						
	OSCC	3	258	3.26 (1.32–8.04)	0.01	60	0.08
	ESCC	5	428	1.48 (0.70–3.12)	0.30	83	<0.001
	LSCC	1	69	2.25 (0.58–8.69)	0.24	—	—
	NPEC	1	75	2.39 (0.57–9.98)	0.23	—	—
	Sample size						
	Large	6	658	1.20 (0.67–2.14)	0.54	78	<0.001
	Small	8	475	3.02 (2.04–4.48)	<0.001	11	0.34
DSS	Overall	5	1182	1.17 (0.74–1.84)	0.50	69	0.01
	Ethnicity						
	Asian	3	969	0.90 (0.61–1.34)	0.61	45	0.16
	Caucasian	2	213	1.76 (1.16–2.68)	0.008	0	0.35
	Sample size						
	Large	2	872	0.82 (0.63–1.06)	0.12	0	0.78
	Small	3	310	1.85 (1.24–2.76)	0.002	0	0.47
	CSC markers						
	Bmi-1	3	310	1.85 (1.24–2.76)	0.002	0	0.47
	Oct-4	1	436	0.79 (0.56–1.12)	0.18	—	—
	Nanog	1	436	0.85 (0.58–1.25)	0.41	—	—
DFS	Overall	3	193	6.04 (3.12–11.69)	<0.001	0	0.65

CSC: cancer stem cell; OS: overall survival; DFS: disease free survival; DSS: disease specific survival; OSCC: oral/oropharyngeal squamous cell carcinoma; ESCC: esophageal squamous cell carcinoma; LSCC: laryngeal squamous cell carcinoma; NPEC: nasopharyngeal carcinoma.
